# Introduction of temporal integration in fourth year MBBS curriculum; Students’ experience

**DOI:** 10.12669/pjms.38.3.4732

**Published:** 2022

**Authors:** Rameen Shahid, Sumera Badar Ehsan, Muhammad Nawaz

**Affiliations:** 1 Rameen Shahid, Final Year MBBS Student, Faisalabad Medical University, Faisalabad, Pakistan; 2 Dr. Sumera Badar Ehsan, Assistant Professor Medical Education, Faisalabad Medical University, Faisalabad, Pakistan; 3 Prof. Dr. Muhammad Nawaz, Department of Ophthalmology, Faisalabad Medical University, Faisalabad, Pakistan

**Keywords:** Professional education, Curriculum, Learning

## Abstract

**Objectives:**

Rapid innovations in medical science have necessitated the development of an advanced medical curriculum. Taking a step towards this, a temporal integrated system and clinic-pathological conferences themes were introduced in a public sector medical university for fourth year MBBS. This study aims to assess students’ perceptions regarding this change.

**Methods:**

A cross sectional study was conducted in a public sector medical university from May 2019 to April 2020. A 17 item pre-validated questionnaire was distributed among all students twice, firstly after completion of first module and then again at the end of the academic year. Results were analyzed using SPSS 21.

**Results:**

There were 265 and 176 participants in the first and second phase of the study respectively. Majority of the students agreed that it was easier to study a topic when temporally integrated. Most commonly reported advantage was better understanding and concept of the theme under study. Suggestions included revision of the schedule regarding allocation of time for different topics and implementation of this curriculum from first year MBBS.

**Conclusion:**

A small step was taken to make improvements in an old traditional method. Students are adapting to this change as they responded positively to various aspects of this methodology. Continuous feedback, evaluation and amendments will help to improve its effectiveness.

## INTRODUCTION

 Rapid innovations in medical science have necessitated the development of a modern teaching curriculum. Globally, such measures have been taken in the form of problem based learning, integrated teaching and self-assisted learning.^1^ Integration is defined as “education that is organized in such a way that it cuts across subject-matter lines, bringing together various aspects of the curriculum into meaningful association to focus upon broad areas of study”.^2^ This way of teaching has been adopted and endorsed by academic councils and medical colleges all over the world.^3^-^5^

 The process of integration is further elaborated into various steps including sharing, nesting and temporal coordination. Temporal coordination, also known as parallel or concurrent teaching, is the scheduling of a lectures in such a way that the same topic is discussed by various disciplines at the same time.^6^ For example, students will discuss the pathology of asthma during pathology lectures and its clinical symptoms, diagnosis and treatment during medicine lessons during the same day or week.

 At present, majority of medical colleges in Pakistan are following the conventional method of teaching that has a discipline based approach towards the subjects being taught. Students are unable to co-relate the information of basic and clinical sciences together and their visions and perspectives of medical knowledge remains confined. Keeping in view the latest trends in medical teaching and bringing a slight change in a rigid traditional system, temporal integration strategy was planned for 4^th^ year with consensus of the relevant heads of department. A timetable was developed to place lectures of similar topics of various disciplines in the same day or week. For example, in week one, students learnt the pathology of atherosclerosis during pathology lectures while its clinical manifestation i.e. coronary artery disease was discussed during medicine lectures in the same week.

 This study aims to explore students experience and perceptions about this pilot project so that things could be improved in future.

## METHODS

 Department heads and medical educationists held meetings to streamline the plan and themes were identified keeping in view the university exam outcomes. As Pathology, community medicine, Ophthalmology, ENT are the examining subjects so the main focus was given to them and medicine and surgical subjects were aligned with the main themes. Timetable for first module is shown in Table-I. Clinicopathological conference were planned in the same way in which students presented the cases which was followed by discussion. It was run for the academic year 2019-20. A total of four modules were planned and each module was of 9 weeks duration.

**Table-I:** Time Table of first module Nov-Dec 2019.

Subject	Week 1	Week 2 & 3	Week 4 & 5	Week 6	Week 7
Pathology	Blood vessel disease	Cardiac pathologies	Respiratory pathologies	CNS pathologies	Breast lesion
Ophthalmology	Retinal vascular diseases	IOP glaucoma	Abnormalities of pupillary reflex and visual pathway	Papilledema, optic atrophy	Retinoblastoma
Otorhinolaryngology	Nasal vascular problems	Trachea, DNS	Airway allergies, rhinitis, sinusitis	CSF rhinorrhea and polyps	Nasal tumors
Community Medicine	Occupational health	Demography	Communicable and non-communicable diseases	Environmental health	Maternal and child health

 This cross sectional study was performed at Faisalabad Medical University from May 19 to April 20 to assess students’ perceptions and thus the effectiveness of temporal integration. Ethical approval was taken on 10^th^ May’ 19 (registration no. 904). A three sectioned questionnaire, using 5-point Likert scale responses and open feedback questions, was developed and piloted. To ensure maximum accuracy, data was collected at two points in time; firstly, at the completion of eight weeks i.e. after first assessment week and secondly at the end of the academic year.

 The first phase was conducted at the end of first assessment week. The questionnaire was distributed to all students present in the lecture hall. A brief overview of the study and questionnaire was given. These students who agreed to participate in the study filled and returned the questionnaires.

 Again in the second phase, the same questionnaire was distributed at the end of the academic year, that is, March 2020. Due to closure of the university because of the Covid-19 pandemic, the questionnaire was sent using google forms on various social media platforms to the students. Again, students were explained the purpose of the questionnaire. SPSS 21 was used for data analysis. Responses in the first and second phases were compared using the Wilcoxon signed-rank test.

## RESULTS

 During the first phase 265 students participated in the study, comprising of 179 (67.5%) females and 86 (32.5%) males. In the second phase there were 176 respondents, 115 (65.3%) females and 76 (34.7%) males. These percentages are concurrent with the gender ratio of our study population i.e. all 4^th^ year MBBS students.

 The overall response regarding the temporally integrated curriculum was positive. About 64.8% respondents in phase 1 and 56.5% students in phase 2 agreed that it was easier to study a theme when taught simultaneously in different subject lectures in the same day or week. Nearly 40% of participants in both phases agreed that it was better than the traditional method of teaching. The most agreed positive aspect was gaining better understanding and concept as indicated by 55.3% and 56.8% students in the first and second phase respectively. However, there were mixed opinions regarding continuation of integrated learning in final year. Except for the views on role of integrated teaching in knowledge retention, there was no significant difference in responses during phase 1 and phase 2. Points were awarded for each response on the Likert scale (1 = strongly disagree, 2 = disagree, 3 = neutral, 4 = agree, 5 = strongly agree). Mean scores are shown in Table-II.

**Table-II:** Comparison of mean scores during first and second phases.

	Mean Score (first phase)	Means score (second phase)	Z	Sig
Temporal coordination provides a better understanding and concept of the topic	3.2±1.25	3.30±1.13	-.83	0.40
It stimulates students’ interest in the subject	3.00±1.10	3.00±1.16	-.195	0.85
Promotes critical thinking	2.93±1.05	3.08±1.08	-.129	0.20
Helps in knowledge retention	3.28±1.10	2.96±1.18	-2.86	0.00
Helps in developing differential diagnosis during ward and out-patient classes	2.94±1.22	3.00±1.12	-.39	0.70
It is easier to grasp a topic when it is taught simultaneously in different subjects	3.59±1.13	3.40±1.67	-1.59	0.11
Temporal coordination is better than the traditional method of teaching?	2.86±1.33	2.84±1.28	-.071	0.94
Would you like to continue with this method in final year MBBS?	2.56±1.53	2.37±1.39	-1.23	0.22
CPC is helpful in attempting OSCEs	2.39±1.16	2.63±1.22	-1.83	0.07
CPC is helpful in attempting long case during clinical examinations	2.72±1.21	2.85±1.19	-1.01	0.31
There should be separate CPC for fourth year students that includes Eye and ENT.	3.25±1.27	3.21±1.20	-.33	0.74
Assessment week helps to study regularly	1.80±1.18	2.01±1.30	-1.66	0.10
It is helpful for preparation of annual professional examinations	2.08±1.40	2.10±1.22	-.55	0.59

 Majority students were not in favor of assessment weeks and preferred a weekly, bimonthly or monthly test system. Popular responses are shown in Table-III and Table-IV. Lack of proper implementation of the timetable and skipping important concepts during lectures were the common problems as reported by 11% and 9% of the participants respectively. Many students mentioned that this method would have been more beneficial if introduced from the beginning as they had difficulty adapting to it.

**Table-III:** Open responses during first phase.

Problems/Suggestions	Other benefits
Regular tests should be taken (50/105)	Relaxation period between assessments (12/105)
Rushing through essential concepts during lectures (18/105)	Proper schedule allows us to plan activities beforehand (5/105)
Difficult to cover two months syllabus on 1 week (11/105)	
Ensure actual implementation of integration (6/105)	
More benefit if implemented from first year (3/105)	

**Table-IV:** Open responses during second phase.

Problems/Suggestions	Other benefits
Regular tests should be taken (17/43)	Proper schedule being implemented rather than haphazard lectures (4/43)
Difficult to sit all tests in a single week (7/43)	Relaxation period between tests allows to spare time for co-curricular activities (3/43)
Lack of proper implementation of schedule (5/43)	
Essential concepts not being covered (4/43)	
Implement from 1^st^ year as it is difficult to adjust now (3/43)	

## DISCUSSION

 Medical teaching and curricula are evolving fast with new methodologies being piloted and adopted for effective learning. Unfortunately, medical schools in Pakistan lag much behind and have deficiencies that were eliminated elsewhere long ago.^7^ These include didactic lectures with learning in fragments. Furthermore, as a part of standardization, accreditation of medical schools by international bodies is becoming mandatory. Educational Committee for Foreign Medical Graduates USA has announced that by 2024, only graduates of accredited medical schools will be eligible for certification.^8^ This makes it necessary to take step by step measures in adopting an updated effective medical education system for our students to stand at par with rest of the world. These include introduction of new teaching elements such as problem based leaning etc.

 Transforming medical education is a long and eventual process. Through integrated teaching, an effort has been made to set the ball rolling. Integrated teaching is considered an effective strategy to bring about coordination in teaching and learning activities.^9^ It has been adopted worldwide to bridge the gap between compartmentalized knowledge and develop a more holistic approach in learning.^10^-^12^ It also gives a better perception of learning environment and helps in knowledge retention.^13^,^14^ A study from a private sector medical university in Pakistan also reports students endorsing integrated teaching.^15^

 Our study aimed to assess the perceptions of medical students regarding the new integrated teaching. This would help in improving the design and overcoming the flaws for a more student-centered curriculum.^16^ Overall, there were mixed responses. Our study showed that almost 55% believed that integrated teaching improved understanding and concept of the topic under study. This percentage is higher than a similar study in Rajasthan, India.^17^ However, other studies have reported more than 90% percent students agreeing to this statement.^18^,^19^ Score for ‘promoting critical thinking’ was less than that of a similar study conducted in India.^10^ Almost 40% students preferred integrated teaching over traditional methods which mirrors the views of students in a similar study.^20^

 Although 67% students in the first and 57% students in the second phase felt that it was easier to grasp a topic, only 37% and 32% in the respective phases wanted to continue integrated teaching in next academic year. The reason for this gap can be explained by responses from the open feedback section. Many students found the assessment pattern tiresome and unsatisfactory. Students in other studies also expressed concerns regarding inability to cope with frequent assessment pattern.^21^,^22^ Some participants reported lack of actual integration of topics, rushing through syllabus contents and skipping important concepts during lectures which is also seen in other studies.^23^ Another view was that it was difficult to adapt to a new learning method and it would have been fruitful if introduced from first year.

 Designing an integrated curriculum is a complex process and involves various challenges.^14^ Evaluation serves as a key tool to overcome the barriers as it will provide important insights and help make appropriate amendments. The concerns of students should be addressed which would pave the way to better outcomes.

### Limitations

Our study also had some limitations. It was based on findings from a single center. Moreover, feedback from faculty was not taken.

## CONCLUSION

Integrated curriculum has its own benefits and challenges. It is a gradual process and its success relies on continuous feedback and modifications. Public sector challenges are unique in so many aspects as changes in decades old curriculum are not readily accepted by faculty and students. Moreover, resources are limited. It is a small effort to bring a minute change so that the path towards integration could be paved with ease.

### Authors’ Contribution:

**RS** did literature search, data analysis, data interpretation and manuscript writing and is responsible for accuracy and integrity of the work.

**SBE** designed the study and questionnaire.

**MN** did study design, data acquisition and revised the manuscript.
